# A Rare Presentation of Acute Kidney Injury in Diffuse Large B-Cell Lymphoma: A Case Report

**DOI:** 10.7759/cureus.45642

**Published:** 2023-09-20

**Authors:** Mahnosh Saleh, Noman Salih, Musa Kakakhel, Izhar Ullah, Hamza Siraj, Muhammad Naveed Tariq, Kamran Ahmad

**Affiliations:** 1 Internal Medicine, Ayub Teaching Hospital, Abbottabad, PAK; 2 Internal Medicine, Hayatabad Medical Complex Peshawar, Peshawar, PAK; 3 Medicine, Hayatabad Medical Complex Peshawar, Peshawar, PAK; 4 Internal Medicine, Khyber Teaching Hospital Peshawar, Peshawar, PAK

**Keywords:** chronic kidney disease (ckd), mediastinal lymphadenopathy, anemia, cervical lymph node biopsy, lymphadenopathy, r-chop chemotherapy, renal infiltration, diagnostic complexities, diffuse large b-cell lymphoma (dlbcl), acute kidney injury (aki)

## Abstract

Acute kidney injury from bilateral renal infiltration is rare in diffuse large B-cell lymphoma (DLBCL). We present the case of a 45-year-old woman with a three-month history of night sweats, weight loss, fever, and fatigue. Clinical evaluation revealed anemia, edema, cervical lymphadenopathy, and elevated blood pressure. Initial lab results indicated severe kidney injury, initially suspected to be chronic kidney disease, later ruled out. Radiological assessments confirmed mediastinal lymphadenopathy. A cervical lymph node biopsy led to a diagnosis of DLBCL. Rituximab, cyclophosphamide, doxorubicin, vincristine, and prednisone chemotherapy improved renal function and hematological parameters. Subsequent staging CT confirmed lymphadenopathy. Close monitoring revealed a complete return to normal renal function after one month. Further follow-up was missed. This case emphasizes diagnostic complexities and the value of a multidisciplinary approach in managing complex clinical presentations.

## Introduction

Malignant lymphoma (ML) is a malignancy originating from the immune system’s infection-fighting cells, specifically the lymphocytes. These lymphocytes are distributed throughout the body, residing in lymph nodes, spleen, thymus, bone marrow, and various other tissues. In the context of lymphoma, there is an aberrant proliferation and uncontrolled growth of these lymphocytes. ML is recognized for its potential to develop in various extra-lymphatic organs, and renal injury is a well-characterized complication in this condition [1]. Irrespective of their rate of proliferation, all MLs have the potential to metastasize to other regions within the lymphatic system if left untreated. Over time, they can also extend their reach to other organs in the body, including, but not limited to, the liver, brain, or bone marrow. Typically, renal damage resulting from lymphoma is attributed to multifaceted factors, including extensive renal infiltration, mechanical compression by the tumor, injury inflicted upon glomerular and tubular structures by tumor-associated proteins, and the potential occurrence of tumor lysis syndrome during chemotherapy treatment [2,3]. Nonetheless, the occurrence of bilateral kidney infiltration by ML is an infrequent phenomenon [4]. Because the renal interstitium lacks lymphatic tissue, the origin of ML cells specifically confined to the kidneys remains an enigma [2,3]. In this report, we present a unique case of acute kidney injury (AKI) attributed to diffuse large B-cell lymphoma (DLBCL), a diagnosis unequivocally ascertained through laboratory investigations. The accuracy of this diagnosis was corroborated via an excisional biopsy of cervical lymph nodes.

## Case presentation

A 45-year-old woman was admitted to our hospital due to a concerning constellation of symptoms that had developed over the course of the past three months. These troubling symptoms encompassed night sweats, unexplained weight loss, persistent high-grade fever, and debilitating fatigue. The gradual onset of these issues raised alarm, especially when she exhibited anuria (lack of urine production) and significant edema in her lower limbs during the past week. Notably, her medical history did not reveal any significant prior medical conditions. The patient presented with signs of anemia and bilateral lower limb edema upon clinical examination. Subsequent evaluation revealed lymphadenopathy in the bilateral cervical region. She had an elevated blood pressure of 155/90 mmHg. The rest of the vital signs were in the normal range. Initial laboratory tests indicated severe kidney injury, as outlined in Table 1. Although there was an initial suspicion of chronic kidney disease (CKD), her previous renal function had been normal. Consequently, a thorough workup for deranged renal function tests (RFTs) was initiated. Urine analysis showed reddish-colored urine and albuminuria. The urine albumin to creatinine ratio was greater than 30. Urine sodium was >40 mmol/L indicating intrarenal involvement. The urine uric acid levels were in the normal range.

**Table 1:** Lab investigations. Hb: hemoglobin; MCV: mean cell volume; WBCs: white blood cells; ALT: alanine aminotransferase; AST: aspartate aminotransferase; ALP: alkaline phosphatase; LDH: lactate dehydrogenase; ESR: erythrocyte sedimentation rate

Test	Reference	Initial value	At discharge	At follow-up
Hb (g/dL)	12.5–17.5	9.1	8.9	9
MCV (fL)	80–100	85	83	85
Platelet (/µL)	150,000–400,000	80,000	84,000	95,400
WBCs (/µL)	4,000–11,000	27,000	23,000	22,100
Na (mmol/L)	135–145	136.6	136	137
K (mmol/L)	3.5–5.1	4.6	4.3	4.4
Cl (mmol/L)	96–112	110	98	104
Creatinine (mg/dL)	0.6–1.1	8.6	5.1	1.3
Urea (mg/dL)	18–45	102	75	50
ALT (U/L)	10–50	47	122	78
ALP (U/L)	<390	145	295	180
AST (U/L)	8–33	23	25	29
Phosphate (mg/dL)	2.8–4.5	5.8	5.3	4.7
LDH (U/L)	105–233	686	315	240
Vitamin D (ng/mL)	20–40	42	-	-
Ferritin (µg/L)	11–307	212	-	-
ESR (mm/hour)	≤20	42	-	-

**Figure 1:**
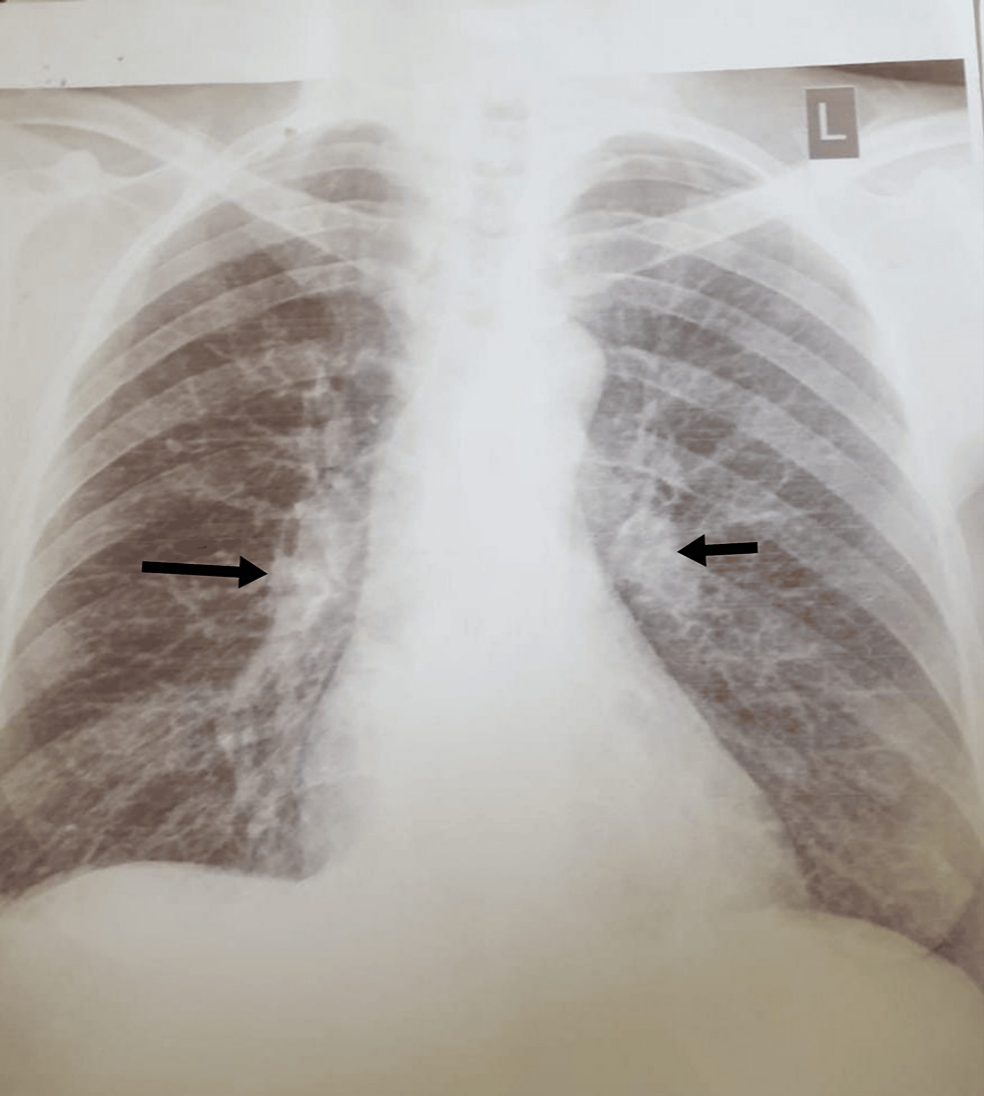
Chest X-ray showing bilateral mediastinal lymphadenopathy.

Chest X-ray depicted bilateral mediastinal lymphadenopathy, as shown in Figure 1, while ultrasonography revealed normal-sized kidneys with no evidence of CKD. Given these findings, malignancy and tuberculosis were considered potential differential diagnoses for both acute kidney injury (AKI) and lymphadenopathy. Thus, further investigations were undertaken to rule out CKD and tuberculosis. Fortunately, all potential causes of AKI and CKD were ruled out, and there was no indication for dialysis.

**Figure 2:**
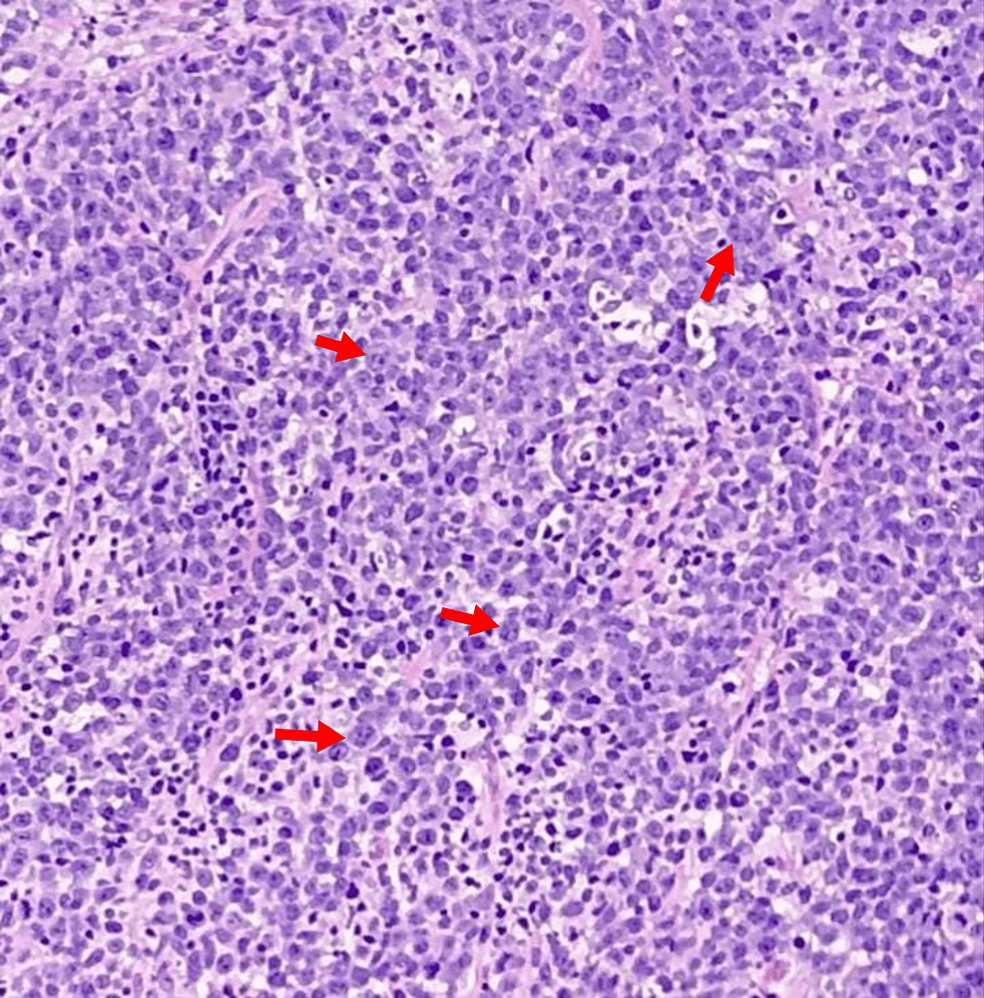
Histopathology slide. Red arrows show diffuse large B cells.

To establish a definitive diagnosis, an excisional biopsy of the cervical lymph node was performed. Histopathological analysis of the sample, as depicted in Figure 2, revealed extensive, diffuse interstitial infiltration by large atypical lymphocytes that tested positive for CD20, CD79a, CD10, multiple myeloma oncogene 1, and B-cell lymphoma 6. This confirmed the diagnosis of DLBCL. While a staging CT scan of the abdomen and pelvis was considered, it was deferred due to abnormal RFTs. A biopsy of the kidney was considered initially but we opted against it and decided to give the patient a trial of fluids and chemotherapy and observe the response. With the DLBCL diagnosis established, the patient initiated treatment with rituximab, cyclophosphamide, doxorubicin, vincristine, and prednisone (R-CHOP) chemotherapy.

**Figure 3:**
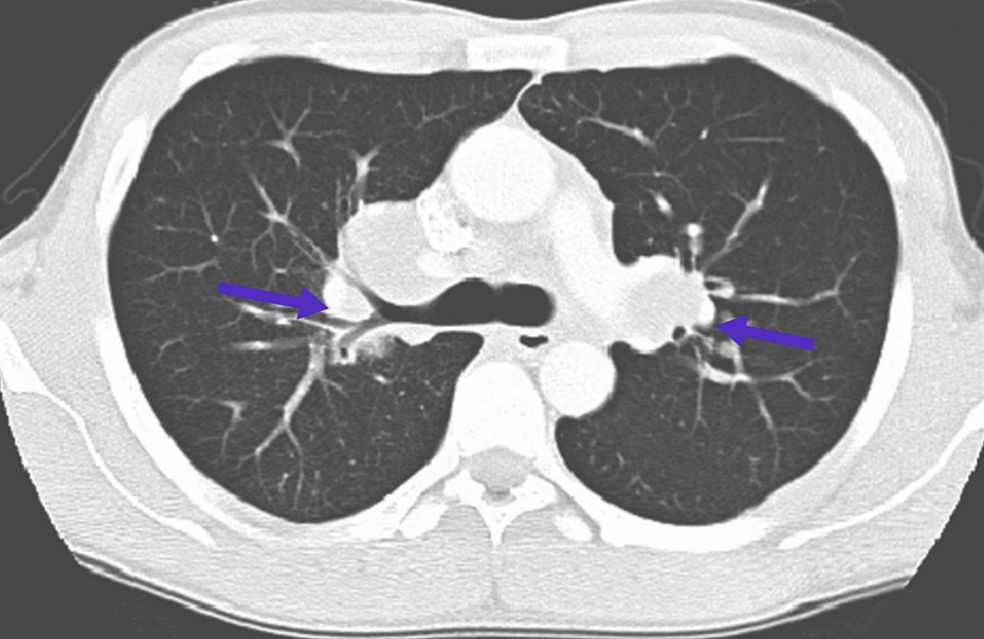
CT of the chest. Blue arrows show bilateral hilar lymphadenopathy.

Over the course of three weeks, her renal function improved secondary to an increase in fluid intake of both IV fluids in the hospital and oral fluids at home which she was advised to take, and her RFTs returned to normal. Her blood tests, including lactate dehydrogenase levels, showed improvement as well. Subsequently, a staging CT scan was performed, as shown in Figure 3, revealing bilateral hilar lymphadenopathy and no evidence of metastasis.

Concerns about potential complications and resource constraints prompted close clinical monitoring of the patient. Over time, her renal function steadily improved, and during a follow-up examination one month later, her renal parameters had returned to normal, marking a significant milestone in her recovery. Unfortunately, the patient did not attend the scheduled follow-up visit and was lost to further follow-up care.

## Discussion

Approximately one-third of DLBCL patients present with renal infiltration on autopsy [4,5]. Bilateral renal involvement is uncommon, making AKI due to diffuse, bilateral tubulointerstitial infiltration a rare initial presentation of lymphoma [6]. Renal involvement in DLBCL can manifest through various direct and indirect effects, including urinary tract obstruction, infiltration, rupture, hypercalcemia, paraproteinemia, amyloidosis, and glomerulonephritis, among others [7].

In our case, while the initial cervical biopsy confirmed extensive infiltration of large atypical lymphocytes expressing specific markers, we opted against renal biopsy due to its invasive nature [8]. Lymphoma cells have the potential to directly impede, infiltrate, or even lead to the rupture of entire segments of the urinary tubule. There is a belief that lymphoma cells that infiltrate these areas can trigger the collapse of the glomerulus [9]. Instead, we initiated a medication trial, allowing us to gauge the patient’s response and determine the appropriate course of treatment.

Bilateral primary renal lymphoma, often associated with AKI, is generally linked with a poor prognosis, with patients surviving less than one year [2,3]. Confirmation of lymphoma cell infiltration into the kidney often occurs postmortem. However, our patient’s case stands out as an antemortem diagnosis was made based on clinical symptoms and response to treatment. Furthermore, the therapeutic effect of R-CHOP therapy was observed based on clinical improvement of the patient.

Renal involvement in ML typically signifies progressive disease [10], often with a high frequency of renal lesions in autopsy cases. However, diagnosing renal involvement in lymphoma before a patient’s demise is challenging, primarily because renal symptoms, as initial symptoms, are exceedingly rare in this context [4]. Treatment can lead to rapid improvements in renal function and tumor size in non-Hodgkin’s lymphoma with renal involvement, as observed in our case, but treatment failure is not uncommon [11].

DLBCL with renal involvement at the time of diagnosis carries a particularly poor prognosis, partly due to the high incidence of central nervous system (CNS) involvement [11]. The mechanisms underlying lymphoma dissemination into the CNS remain unclear, although certain genes and adhesion molecules on the surface of non-Hodgkin’s lymphoma cells have been associated with extranodal involvement, tumor aggressiveness, and poorer outcomes [11,12]. Strategies to prevent CNS relapse in patients with renal involvement, including the use of rituximab, have shown limited effectiveness. A previous report highlighted renal involvement as an independent risk factor for CNS relapse in DLBCL patients in both pre- and post-rituximab treatment eras [13].

Renal infiltration of DLBCL is generally considered a secondary process, indicating progressive disease. Given its association with CNS relapse, early evaluation of the CNS is crucial in this patient population.

## Conclusions

In summary, this case posed an initial diagnostic uncertainty, with a 45-year-old woman experiencing troubling symptoms over months. Severe kidney injury was first suspected as CKD but was ruled out. Further investigations revealed DLBCL, confirmed by lymph node biopsy. Treatment with R-CHOP chemotherapy resulted in significant renal and overall clinical improvement. Although the staging CT was delayed due to renal issues, the patient showed positive responses. However, the patient’s decision to discontinue follow-up care and be lost to further assessment underscores the need for vigilant monitoring in complex cases. This case underscores the importance of a multidisciplinary approach, thorough investigations, and prompt intervention in managing such challenging presentations.
